# An Anomalous Right Coronary Artery Originating from the Left Anterior Descending Artery, a Case Report of Successful Percutaneous Coronary Intervention

**DOI:** 10.15388/Amed.2025.32.2.7

**Published:** 2025-12-30

**Authors:** Ebrahim Nematipour, Arash Shekari, Shapour Shirani, Seyyed Mojtaba Ghorashi

**Affiliations:** 1 Department of Cardiovascular Research, Tehran Heart Center, Tehran University of Medical Sciences, Tehran, Iran; 2 Department of Cardiovascular Research, Tehran Heart Center, Tehran University of Medical Sciences, Tehran, Iran; 3 Department of Cardiovascular Research, Tehran Heart Center, Tehran University of Medical Sciences, Tehran, Iran; 4 Department of Cardiovascular Research, Tehran Heart Center, Tehran University of Medical Sciences, Tehran, Iran

**Keywords:** percutaneous coronary intervention, coronary vessel anomalies, cardiovascular abnormalities, perkutaninė koronarinė intervencija, koronarinių kraujagyslių anomalijos, širdies ir kraujagyslių sistemos anomalijos

## Abstract

**Background:**

An anomalous right coronary artery (RCA) originating from the left anterior descending artery (LAD) is a rare subtype of single coronary artery ostium. Revascularization in such cases is challenging due to the large feeding territory or the potential for compression by an adjacent vessel.

**Case description:**

We report the case of a 57-year-old woman who presented to our hospital with exertional chest pain and dyspnea. An anomalous RCA was identified, originating from the mid-portion of the LAD. Coronary angiography and coronary multi-detector computed tomography revealed a significant stenosis at the LAD just proximal to the RCA bifurcation. A successful percutaneous coronary intervention was performed to revascularize the LAD stenosis. The patient was discharged in good general condition two days later.

**Conclusions:**

Despite the rarity of coronary anomalies, future studies could be undertaken to assess the potential benefits of screening, particularly in specific populations such as professional athletes.

## Abstract (it)

**Įvadas:**

Pakitusi dešinioji vainikinė arterija (RCA), prasidedanti iš kairiosios priekinės nusileidžiančios arterijos (LAD), yra retas vienos vainikinės arterijos ostiumo potipis. Tokiais atvejais revaskuliarizacija yra sudėtinga dėl didelio aprūpinimo teritorijos arba galimo gretimų kraujagyslių suspaudimo.

**Atvejo aprašymas:**

Pateikiame 57 metų moters, kuri kreipėsi į mūsų ligoninę dėl krūtinės skausmo ir dusulio fizinio krūvio metu, atvejį. Buvo nustatyta dešiniosios vainikinės arterijos (RCA), prasidedančios iš kairiosios priekinės nusileidžiančios arterijos (LAD) dalies, anomalija. Koronarinė angiografija ir koronarinė daugiadetektorių kompiuterinė tomografija atskleidė reikšmingą LAD stenozę ties RCA bifurkacija. Perkutaninė koronarinė intervencija, siekiant revaskuliarizuoti LAD stenozę, buvo sėkmingai atlikta. Po dviejų dienų pacientė išrašyta iš ligoninės, bendra jos būklė buvo gera.

**Išvados:**

Nepaisant to, kad koronarinės anomalijos retos, ateityje galėtų būti atliekami tyrimai, siekiant įvertinti potencialią atrankinės patikros naudą, ypač tam tikrų populiacijų, pavyzdžiui, profesionalių sportininkų.

## Introduction

A *Single Coronary Artery Ostium* (SCAO) is an important type of coronary artery anomaly. It is usually an incidental finding [1]. We present a patient with an anomalous *Right Coronary Artery* (RCA) originating from the *Left Anterior Descending* artery (LAD), with significant stenosis in the mid-portion of the LAD just proximal to the RCA origin. This vascular anomaly is rare; an abnormal origin of the RCA is not the most common type of coronary anomaly, and its origin from the LAD is also an uncommon variant among RCA anomalies. Meanwhile, its pathologies are associated with serious complications [1].

In the present study, we describe the patient’s characteristics and treatment course, and discuss the nature of this coronary anomaly as well as its possible complications.

## Case report

A 57-year-old woman presented to our center with exertional chest pain (functional class II; The *New York Heart Association* (NYHA) classification) and dyspnea on exertion (functional class II; NYHA classification). She also had diabetes mellitus, hypertension, dyslipidemia, and diabetic retinopathy. Informed consent was obtained from the patient. Echocardiography showed a normal ejection fraction, and valvular heart disease was excluded. The patient’s pre-admission medications included aspirin, atorvastatin, valsartan, gliclazide, and a combination of empagliflozin/metformin. The patient’s laboratory data are presented in Table 1.

**Table 1:** Laboratory data of the patient

Parameter	Result	Laboratory reference range	Parameter	Result	Laboratory reference range
FBS	201 mg/dL	70-100 mg/dL	Platelet count	215000 /µL	150000-450000 /µL
HbA1c	7.43%	non-diabetic: 4-6% goal: 6-6.5% good: 6.5-8% uncontrolled: > 8%	TSH	1.95 mIU/mL	0.27-4.2 mIU/mL
Triglyceride	134 mg/dL	< 150 mg/dL	Sodium (Na)	136.7 mEq/L	135-145 mEq/L
Total cholesterol	175 mg/dL	< 200 mg/dL	Potassium (K)	4.9 mEq/L	3.5-5 mEq/L
HDL cholesterol	56 mg/dL	low risk: >60 mg/dL borderline: 35-60 mg/dL high risk: <35 mg/dL	ALT	16 IU/L	< 33 IU/L
LDL cholesterol	95 mg/dL	< 130 mg/dL	AST	13 IU/L	< 32 IU/L
WBC	6000 /µL	4000-10000 /µL	ALP	99 IU/L	< 240 IU/L
Hemoglobin	11.5 g/dL	11-16 g/dL	Creatinine	1.2 mg/dL	0.5-1.2 mg/dL
MCV	88.1 fL	80-96 fL	Uric acid	5.7 mg/dL	3-7 mg/dL

Abbreviations: FBS, fasting blood sugar; HbA1c, Hemoglobin A1c; HDL, high-density lipoprotein; LDL, low-density lipoprotein; WBC, white blood cells; MCV, mean corpuscular volume; TSH, thyroid stimulating hormone; ALT, alanine aminotransferase; AST, aspartate aminotransferase; ALP, alkaline phosphatase

**Figure 1:**
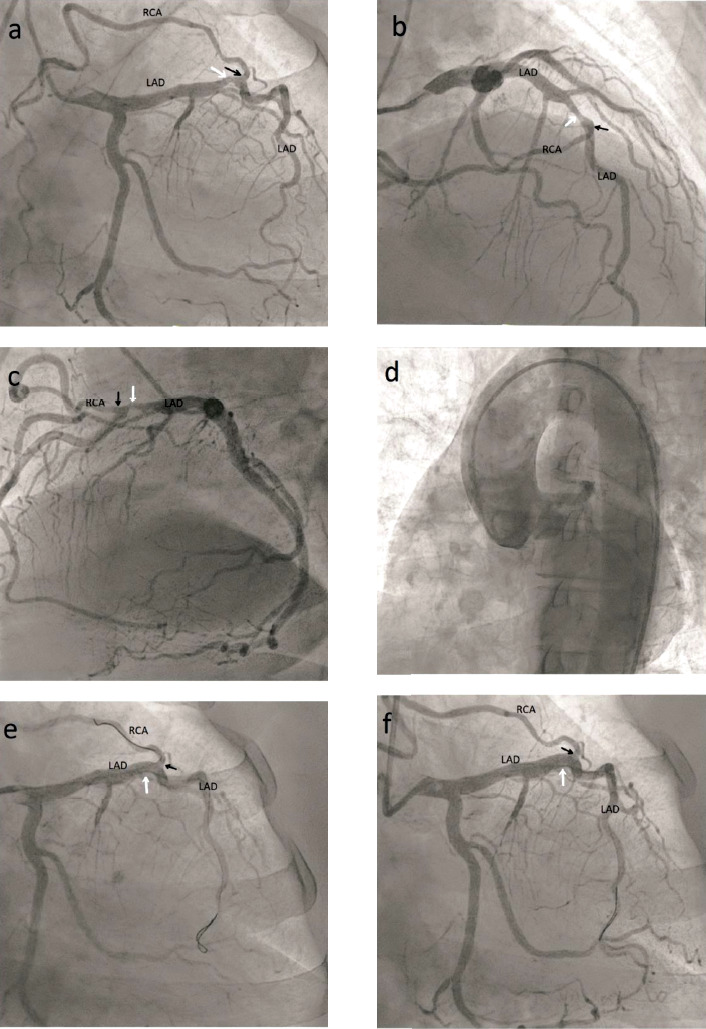
**(a, b, c)** The right coronary artery (RCA) originates from the mid-left anterior descending artery (LAD), just distal to the lesion. **(d)** Aortic root injection shows no origin of the RCA from the right coronary cusp. **(e, f)** Successful percutaneous coronary intervention (PCI) results are demonstrated, with and without the side branch wire, respectively. White arrows indicate the site of stenosis in the LAD before and after PCI, whereas black arrows indicate the anomalous origin of the RCA from the LAD.

Coronary angiography showed an anomalous RCA originating from the mid-portion of the LAD (Figure 1). A significant stenosis was observed at the mid-portion of the LAD just proximal to the origin of the RCA.

**Figure 2:**
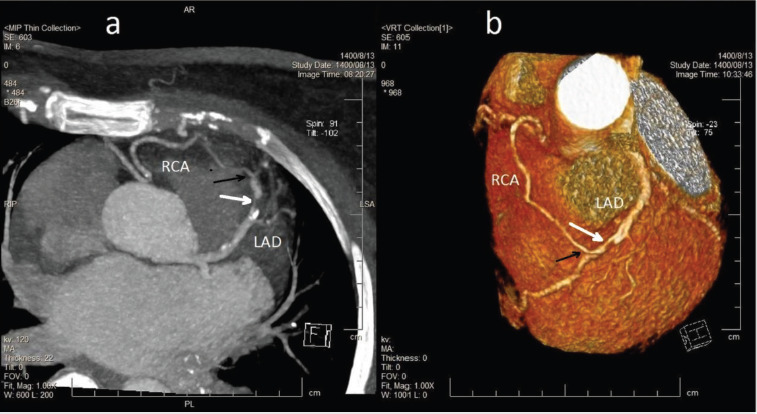
**(a)** Maximum intensity projection and **(b)** volume rendering technique reconstruction images from coronary *Multi-Detector Computed Tomography* (MDCT). Coronary MDCT reveals the anomalous origin of the right coronary artery (RCA) from the mid-left anterior descending artery (LAD), distal to a stenotic segment. Stenosis was severe (70–99%) in the LAD. White arrows indicate the site of stenosis in the LAD, whereas black arrows point to the anomalous origin of the RCA from the LAD.

Coronary multi-detector computed tomography was done for better evaluation of the coronary anomaly, which confirmed an anomalous RCA originating from the mid-portion of the LAD (Figure 2). In addition, the anomalous RCA traversed anterior to the *Right Ventricular Outflow Tract* (RVOT). A significant stenosis in the LAD just before the RCA bifurcation was confirmed. The calcium score was calculated by using the Agatston scoring system, and the calcium score value was 53. A *Percutaneous Coronary Intervention* (PCI) was performed for revascularization of the LAD stenosis because of the patient’s symptoms.

The patient’s cardiovascular diagnosis was coronary artery disease, characterized by severe stenosis of 70 to 99 percent in the mid-LAD. According to the 2022 coronary artery disease-reporting and data system (CAD-RADS) 2.0 classification, this finding corresponds to a CAD-RADS score of 4A/P1 [2].

The trans-radial coronary intervention was performed by using an XB 3.5 6 French guiding catheter engaged in the left coronary artery. Then, a guidewire (ASAHI Soft 0.014) was placed in the LAD. Another guidewire (ASAHI SION blue 0.014) was placed in the RCA as a side branch to support the artery. Then, a stent (Ultimaster 3.5 × 24 millimeters (mm)) was placed in the stenotic segment directly, and the stent balloon was inflated to a pressure of 8 atmospheres (atm). A 4 × 12 mm non-compliant Sapphire balloon was used for post-dilatation, and was inflated to a pressure of 18 atm. The result was a successful PCI without any complications, and no stenosis remained. The patient was discharged after two days in good general condition.

## Literature review and discussion

The estimated incidence of coronary artery anomalies is reported to be about 1.3% [1]. Single coronary artery ostium is a type of coronary artery anomaly. In SCAO, there is a common single ostium for all coronary artery branches and at least one of the LAD, right coronary, or left circumflex arteries has an ectopic origin from another artery [3]. The prevalence of an anomalous RCA origin from LAD or left circumflex or left sinus of Valsalva in the angiography of all patients was presented as 0.13%, 0.18%, and 0.036%, in the studies by Quali et al., Aidinlar et al., and Yuksel et al., respectively [4]. Its prevalence in the general population remains unknown.

Regarding the embryology of coronary arteries, the ostium and coronary stem develop between embryonic days 37 and 47, the main coronary arteries develop between embryonic days 47 and 56, and the arterial branches are organized between embryonic days 54 and 70 [3].

Vascular smooth muscle cell precursors and neural crest cardiac cells play an essential role in developing coronary arteries. *Vascular Endothelial Growth Factor* (VEGF) plays a critical role in the development of the coronary artery ostium. The absence of the VEGF-B isoform is considered a crucial factor in the inhibition of ostial formation [3].

An anomalous RCA originating from the LAD is a very rare subtype of SCAO [5]. Such an anomalous RCA commonly traverses anterior to the RVOT and usually originates from the mid-portion of the LAD, which is defined as an RCA originating after the first septal perforator branch [1,5]. Approximately 97% of patients with this anomaly have no structural heart disease [6].

There is controversy regarding the clinical significance of this type of coronary artery anomaly [6]. Some authors have reported that accelerated atherosclerosis may occur in these coronary arteries [4]. Moreover, revascularization of this type of anomaly can be challenging due to factors such as a large feeding territory, a possible acute bifurcation angle that may reduce the flow velocity, compression by an adjacent vessel, and an abnormal orifice [5]. Meanwhile, *Myocardial Infarction* (MI) associated with this type of SCAO is rare [6].

These patients may be asymptomatic or may be admitted with MI in several territories with or without atherosclerosis [6]. The course of the RCA was anterior to the RVOT in the case reported by Salih et al., similar to our case [1]. It seems that the malignant course of an anomalous RCA increases the possibility of sudden cardiac death, and it is seen less commonly in elderly patients with acute coronary syndrome or MI symptoms [5]. In general, approximately 15% of patients with an anomalous RCA were admitted with MI without atherosclerosis [7].

In a review article published in 2009, only 36 cases were reported with this type of anomaly [6]. Most reported cases were identified after the age of 50. Fourteen out of 36 patients required revascularization, of whom, only four patients underwent PCI [6]. In addition, only 23 cases were reported from 2009 to 2022, of whom, seven patients underwent PCI. Medical treatment, PCI, and surgery were used in patients with an anomalous RCA originating from the LAD [6].

Similar to our case, other studies have used coronary *Computed Tomography Angiography* (CTA) for a more detailed examination of the anatomy of the anomalous artery, which can affect the treatment approach [5]. Cardiac *Magnetic Resonance Imaging* (MRI) is probably just as useful.

In Wilson et al.’s study, as well as in our study, the RCA originated from the LAD, and stenosis occurred in the LAD proximal to the RCA origin. The patient had symptoms of ischemia and, unlike our patient who was treated with PCI and a stent, the patient discussed by Wilson et al. was treated with coronary artery bypass grafting [6]. The choice of the type of treatment for pathologies of coronary anomalies also depends on the patient’s condition, the type of anomaly, and the severity of vascular involvement.

Based on PCI and stenting for anomalous vessels in a few reported cases, including ours, no excessive complications or treatment failures have generally been observed.

The association between coronary anomalies and sudden cardiac death in professional athletes has also been highlighted [4,8]. In one study, approximately 13% of such deaths were attributed to coronary anomalies, which were typically not previously screened for [8].

## Conclusions

The presently mentioned case represents another successful PCI treatment in a patient with complicated SCAO and stenosis near the bifurcation. Despite the rarity of coronary anomalies, future studies could explore the benefits of screening, for example, by using cardiac MRI or coronary CTA, particularly in specific populations such as professional athletes, so that to identify anomalies associated with a higher risk of complications.
